# Role and Therapeutic Targeting of the PI3K/Akt/mTOR Signaling Pathway in Skin Cancer: A Review of Current Status and Future Trends on Natural and Synthetic Agents Therapy

**DOI:** 10.3390/cells8080803

**Published:** 2019-07-31

**Authors:** Jean Christopher Chamcheu, Tithi Roy, Mohammad Burhan Uddin, Sergette Banang-Mbeumi, Roxane-Cherille N. Chamcheu, Anthony L. Walker, Yong-Yu Liu, Shile Huang

**Affiliations:** 1 College of Pharmacy, University of Louisiana at Monroe, Monroe, LA 71209-0497, USA; 2 Division for Research and Innovation, POHOFI Inc., P.O. Box 44067, Madison, WI 53744, USA; 3 School of Nursing and Allied Health Sciences, Louisiana Delta Community College, Monroe, LA 71203, USA; 4 Department of Biochemistry and Molecular Biology, Louisiana State University Health Sciences Center, 1501 Kings Highway, Shreveport, LA 71130-3932, USA; 5 Feist-Weiller Cancer Center, Louisiana State University Health Sciences Center, Shreveport, LA 71130-3932, USA

**Keywords:** PI3K, Akt, mTOR, skin cancers, phytochemicals, melanoma, basal cell carcinoma, squamous cell carcinoma, Merkel cell carcinoma, targeted therapy

## Abstract

The mammalian or mechanistic target of rapamycin (mTOR) and associated phosphatidyl-inositiol 3-kinase (PI3K)/protein kinase B (Akt) pathways regulate cell growth, differentiation, migration, and survival, as well as angiogenesis and metabolism. Dysregulation of these pathways is frequently associated with genetic/epigenetic alterations and predicts poor treatment outcomes in a variety of human cancers including cutaneous malignancies like melanoma and non-melanoma skin cancers. Recently, the enhanced understanding of the molecular and genetic basis of skin dysfunction in patients with skin cancers has provided a strong basis for the development of novel therapeutic strategies for these obdurate groups of skin cancers. This review summarizes recent advances in the roles of PI3K/Akt/mTOR and their targets in the development and progression of a broad spectrum of cutaneous cancers and discusses the current progress in preclinical and clinical studies for the development of PI3K/Akt/mTOR targeted therapies with nutraceuticals and synthetic small molecule inhibitors.

## 1. Introduction

### 1.1. Structure and Function of the Human Skin

**Figure 1:**
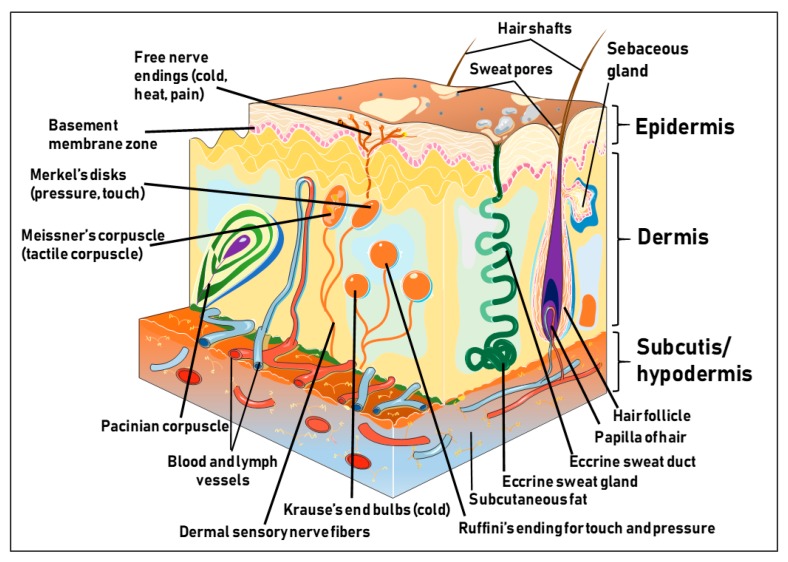
Schematic representation of the cross section of the skin. The skin is composed of epidermis, dermis, and hypodermis. The dermis supports and provides nourishment to the overlying epidermis through its constituent blood and lymphatic vessels. The skin also comprises sensory elements, various resident cell types like fibroblasts, macrophages, and lymphocytes. The ectodermally derived appendage such as sweat glands, sebaceous glands, and hair follicles arises as an invagination of the epidermis into the dermis. The epidermal stem cells are found in the bulge region of the hair follicles, in the basal layer of the interfollicular epidermis, and to some extent the sebaceous glands.

The human skin is the outermost and largest organ constituting about 20% of the total body weight and measures a total surface area of approximately 2 m^2^ in an adult human [1]. The skin is strategically positioned at the interface between the internal and external worlds. While the skin looks simple on the surface, underneath it presents a unique and complex biochemical structure with properties that confer multiple functions vis-à-vis shaping the body [2]. The skin provides a major dynamic, mechanical, and physical defensive barrier against external insults such as toxic chemical agents, infectious microorganisms, ultraviolet (UV) radiation, as well as mechanical stressors [2,3,4,5]. In addition to barrier function, the skin also regulates the inward and outward passages of body fluids, including water, electrolytes, and various substances; mediates immune- and thermo-regulatory responses; coordinates sensory perception; and serves as a metabolic sink for the storage of energy in the hypodermis [6,7,8]. Histologically, the skin is mainly composed of three distinct and unifying tissue components. The first one is the epidermis, an ectodermally derived outermost, non-vascularized, stratified, keratinizing squamous epithelium, with multiple layers ranging in thickness between 75 and 150 μm (interfollicular), and up to 600 μm (the palms and soles). The second one is the dermis, a mesodermally derived, dense collagen, and elastin-rich fibrous connective tissue inundated with blood and lymph vessels, nerve elements, and embedded with disseminated cells including fibroblasts, mast cells, macrophages, and lymphocytes. The third one is the hypodermis or subcutaneous tissue, which underlies the dermis, and mainly consists of fatty or adipose tissues that are regularly crisscrossed by blood vessels [7] (Figure 1).

### 1.2. The Epidermis

**Figure 2:**
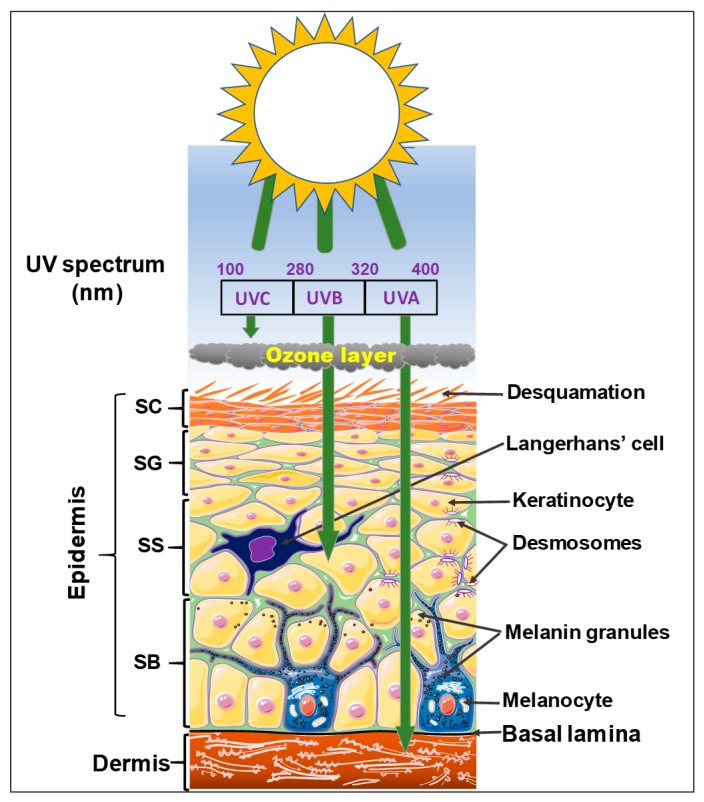
Schematic representation of the structure of the epidermis and papillary dermis. This stratified epithelium consists of basal, spinous, granular, lucidum, and stratum corneum layers. The basal layer contains the stem cells that sit or attach to the basal lamina via hemidesmosomes. In normal epidermis, basal keratinocytes express K5, K14, and integrins. The suprabasal and differentiating layers of keratinocytes are committed to differentiation, expressing K1, K10, and other markers, like filaggrin and loricrin. Other resident epidermal cell types include Melanocytes, Langerhans cells, and Merkel cells. The ultraviolet rays (UVR) from the sun penetrate the atmosphere and the different layers of the skin. UVC with the greatest energy photons (shorter wavelengths) is totally absorbed by the ozone layer while UVB with intermediate energy photons (wavelengths) that damage DNA in epidermal cells penetrates the upper layers of the epidermis. UVA with low energy photons (longer wavelengths) penetrates deeper into the upper dermis causing damage to collagen and elastic tissue, even skin cancers and other skin manifestations.

The epidermis, an outermost skin tissue and a semi-permeable covering, functions as the major contact point of the body with the external environment. In adults, the epidermis is mainly composed of over 90% keratinocytes, epidermal cells that synthesize keratin, a diverse group of cytoskeletal scaffolding proteins that form 10–12 nm intermediate filament networks at different stages of cornification with functions that determine the condition of the epidermis and make the skin impermeable [7]. Under normal physiologic condition, there is a balance between the renewal and the death of the keratinocytes in maintaining homeostasis [9,10]. There exist other cell types housed in the viable epidermis including melanocytes, Langerhans cells, and Merkel cells, which equally contribute to ensuring skin homeostasis. Melanocytes are the skin pigment producing cells located at the dermal-epidermis junction and hair follicles, which synthesize melanin within melanosomes, and ensure skin pigmentation and photo-protection and other physiological regulatory and protective action [11,12,13,14,15]. Langerhans cells are antigen-presenting cells responsible for immune function of the skin and provide protection against external invading substances and microorganisms [7,16,17,18,19,20,21]. Merkel cells are oval-shaped mechanoreceptors essential for light touch sensation [18]. On the basis of the skin site, histological skin cross-section reveals that the epidermis is divided into four (hairy or interfollicular skin) or five (palmo-plantar or glabrous skin such as soles and palms) distinct cell layers or strata characterized by different stages of keratinocytes maturation (Figure 2) [9,22]. From inward-out to the skin surface, the deepest layer of the epidermis is the basal or germinative cell layer (stratum basale; SB), which harbors resident stem cells and their progenitor transient amplifying (mitotically active) keratinocytes. There exist three different epidermal keratinocyte stem cell pools located at: 1) the basal compartment, 2) the tip of the dermal papillae, and 3) the hair follicle bulge that is attached to the basement membrane zone (BMZ) via hemidesmosomal protein complexes [6,22,23,24,25]. Therefore, the skin serves as a local reservoir of various adult/multipotent stem cell populations, both within the basal epidermal and dermal tissue compartments [9,24,26]. Epidermal homeostasis is maintained by these multipotent stem cells from hair follicle to non-follicular skin, which possess the capacity to regenerate and differentiate into multiple cell lineages, for epidermal, hair/non-hair follicles, and sebaceous glands [27]. During mitosis, a sister keratinocyte stem cell maintains the stem cell pool, while the other pool of daughter keratinocytes divides asymmetrically into progenitor keratinocytes committed to terminal differentiation [9,25,27]. Upon commitment to differentiation, the progenitor basal keratinocytes proliferate, detach, and move towards the skin surface by sequentially developing into the spinous, granular, and cornified layers in a cornification process and subsequently are desquamated [9,28]. During cornification, as basal keratinocytes expressing keratin 5 (K5), keratin 14 (K14), and integrins detach and transition upward, several biochemical processes lead to gene expression of keratinocyte differentiation-related markers such as keratins 1 (K1), 10 (K10), filaggrin, involucrin, loricrin, and transglutaminase [28,29]. Overlying the basal layer is the spinous or prickle cell layer (stratum spinosum; SS) that is several layers thick and is characterized by increased numbers of desmosomes. Sitting on this layer is the granular cell layer (stratum granulosum; SG) that comprises about 3–5 cell layers encircling lamellar bodies and keratohyalin granules [29]). Here, the cells gradually flatten and collapse, which is associated with initiation of nuclear and other organelle degradation, and active lipid and protein secretion. In palmoplantar skin sites, the granular layer is overlaid by the clear layer (stratum lucidum, SL), which corresponds to a transition phase between the granular layer and the stratum corneum, but the SL is absent in interfollicular epidermis as discussed above. Finally, the cornified or horny cell layer (stratum corneum, SC) is the outermost layer of the epidermis that is comprised of 3–10 layers of flattened corneocytes ranging 10–30 μm thick, as well as intercellular lipids/protein complexes on the skin surface and provides the vital skin barrier against water loss and external insults [7,30]. The process of cornification culminates in the formation of the stratum corneum (SC), the last line of defense from the external environment, which is composed of corneocytes, dead keratinocytes containing a highly specialized protein and lipid matrix and forms a vital part of the skin surface that provides the barrier [7,30]. Corneocytes are ultimately lost through desquamation and replaced by newly differentiated cells, a process resulting in the regeneration of this tissue every 6–8 weeks in humans and 8–10 days in mice [31]. By readily diffusing through the intercellular layers, the SC may allow the transportation of some small, lipid-soluble compounds from the surface inwards into the skin, and hence, the integrity of the skin as a dynamic organ is maintained through epidermal homeostasis. The balance between epidermal keratinocyte proliferation and differentiation is tightly regulated, and deregulation of this balance is regarded as the cause of diverse skin pathologies including cutaneous cancers, and inflammatory skin disease.

### 1.3. The Dermis

Beneath the epidermis sits the mesodermally derived dermis (intermediate skin layer), a thick layer of dense connective tissue mostly consisting of the ground substance or extra cellular matrix (ECM) particularly made up of collagen, elastin, fibrillin, and glycoproteins (non-structural) that give the skin its suppleness and mechanical strength [7,32,33]. The dermis is comprised of two major layers: a) the papillary dermis (superficial dermis), an intermediate layer rich in nerve endings, which is separated from the epidermis by the dermal-epidermal junction (Figure 1); and b) the reticular dermis (deep and medium dermis), a dense connective tissue composed of a network of elastic fibers. The dermis harbors blood vessels, hair follicles, nerve endings, sweat, and sebaceous glands that support and nourish the epidermis, and protects the vascular network and nerve fibers (Figure 1). The dermis also harbors an abundance of different resident cell types, including fibroblasts that synthesize collagen/ECM essential for tissue elasticity, and histiocytes such as macrophages, lymphocytes and mast cells important in skin immune response.

### 1.4. The Hypodermis

Underneath the dermis lies the hypodermis or subcutaneous tissue (or subcutis) containing adipose tissue, blood vessels, nerves, and sometimes invaginations of epidermal appendages such as sweat glands, sebaceous glands, and hair follicles, enabling the hypodermis to insulate the body and serve as an energy reservoir as well [1,6] (Figure 1). As shown in (Figure 1), skin derivatives or appendages including the hair follicles, nails, sebaceous, sweat, and apocrine glands, also derived from embryonic ectoderm are absent in palmoplantar or load bearing skin sites such as the palms and soles [6]. Although the skin varies in thickness based on the anatomical site, age, and the presence and density of derivatives, the basic structure is maintained at all body sites; all three skin tissue compartments interact and communicate with each other through the secretion of immune-mediators, extracellular matrix proteins, growth factors and hormones [6,34]. Being a functional barrier, the skin is constantly in direct contact with the outside environment. Since healthy skin is a major component of our physical appearance, skin also plays an important role in our social and sexual relationships. Perturbation in the intricate organization due to acquired or inherited factors leads to several cutaneous diseases, most of which are chronic and recalcitrant to treatment.

## 2. Risk Factors Associated with Cutaneous Carcinogenesis

Two major risk factors are associated with the pathophysiology of many cutaneous carcinogenesis, including environmental (also termed modifiable) and genetic (also termed non-modifiable) risk factors [18,35]. The most common environmental risk factor or trigger for almost all skin cancer types is exposure to ultraviolet (UV) radiation [36], which can damage DNA in skin cells like keratinocytes and melanocytes, leading to tanned and sunburned skin [35]. As depicted in (Figure 2), UV radiation forms a portion of the electromagnetic (EM) spectrum that reaches the Earth from the sun. UV rays are situated between X-rays and visible light, and contain three major types, UVA, UVB, and UVC, at varying wavelengths ranging from 100 to 400 nanometers (nm) with varied skin penetrating properties [37]. UVA rays have the longest wavelengths (320–400 nm), followed by UVB rays with medium wavelengths (290–320 nm), and then UVC rays have the shortest wavelengths (100–280 nm). UVA rays are not absorbed by the atmosphere (Earth’s ozone layer), so they are transmitted through and can penetrate deep into the middle layer of the skin via the basement membrane, where the melanocytes reside to the superficial dermis [38]. UVB rays are almost totally absorbed by the epidermis (Figure 2). UVC rays are mostly absorbed by the ozone layer and the atmosphere, to some extent, that is often dependent on the climatic conditions [36,39]. Thus, most of the UV rays that come in contact with the skin are UVA with a small amount of UVB [38]. Both UVA and UVB exposures can result in a tanned skin appearance [37], and overexposure to UVB radiation causes erythema, swelling, and pain, the characteristic signs of sunburn, which generally take several hours to develop.

Incident UV rays unto the skin can intermingle with numerous light-emitting skin layer specific molecules to elicit both desirable and undesirable effects, contingent upon the UV rays’ exposure, sources, and wavelength. Desirable effects include priming the skin to synthesize vitamin D precursor as well as their intake, in view of treating various cutaneous diseases including cancers [40,41,42]. Undesirable effects of UV rays in skin include allergic and inflammatory diseases, immunosuppression, photo-aging, oxidative stress, carcinogenesis, and increased drug sensitivity [35,43,44,45]. The molecular mechanism of UV-induced skin cancers is associated with eliciting increased DNA damage signals, e.g., activation of the p53 pathway and induction of the apoptotic pathway, which profoundly alter cell physiology to mediate cell cycle arrest and activate DNA repair [44,45]. Interestingly, exposure of human keratinocytes to UVA and UVB results in activation of the phosphatidyl-inositiol 3-kinase (PI3K) as well as phosphorylation of Akt at S473 by UVB and at Thr308 by UVA as well as increased phosphorylation of the mammalian or mechanistic target of rapamycin (mTOR) and p70 S6 kinase 1 (S6K1). Rapamycin pretreatment has been shown to suppress the expression of phosphorylated S6K1 upon exposure to UV radiation, and the silencing of Akt had no effect on its expression, an indication that exposure to UV radiation can activate the PI3K/Akt/mTOR-S6K1 pathway [46].

## 3. The PI3K/Akt/Mtor Signaling and Interrelations in Tissue Development

In multicellular organisms, several signaling pathways are associated with the regulation of gene expressions, thus contributing to the organized complex physiological processes critically involved in skin cell growth, proliferation, survival, and differentiation, as well as skin tissue development [47,48]. Consequently, alterations in these pathways can modulate protein synthesis, negatively impact skin cell growth and proliferation, and result in phenotypically diverse skin diseases [47,48,49]. Knowledge of the intracellular signals and mechanisms through which cells receive and integrate extracellular cues is important for the diagnosis and the development of novel and well-targeted therapeutic regimen for ensuing cutaneous malignancies. Amongst various signal transduction pathways, the PI3K/Akt/mTOR pathways [50,51] are the hub involved in a variety of physiologic functions linking growth factors, nutrients, and energy availability to lipid and protein synthesis, metabolism, cell growth, proliferation, survival, apoptosis, angiogenesis, and tissue development [52,53]. These pathways and associated components have been frequently observed to be deregulated in diverse cancers including the melanoma and non-melanoma skin cancers and are emerging as clinically relevant therapeutic targets [52,53].

## 4. Structure and Function of the mTOR Pathway

**Figure 3:**
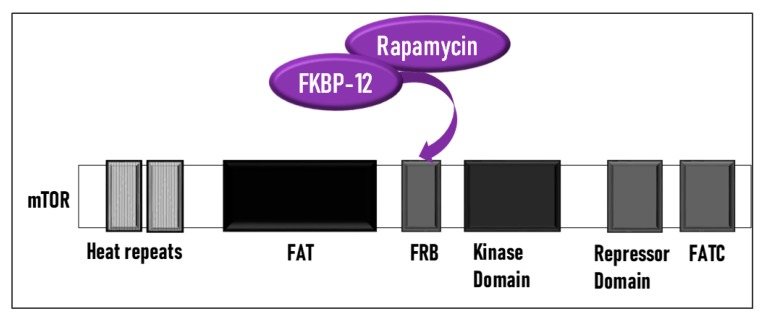
Schematic of the domain structure of mTOR showing the *N*-terminus with two tandem HEAT repeats, followed by a FAT domain (domain shared by PI3K-related protein kinases (PIKK) family members), an FRB domain (FKBP-12-rapamycin-binding site), a kinase catalytic domain, a repressor domain (RD), and a FAT C terminus domain located at the *C*-terminus of the protein. The FRB domain forms a deep hydrophobic cleft that serves as the high-affinity binding site for the inhibitory complex FKBP12-rapamycin [52].

**Figure 4:**
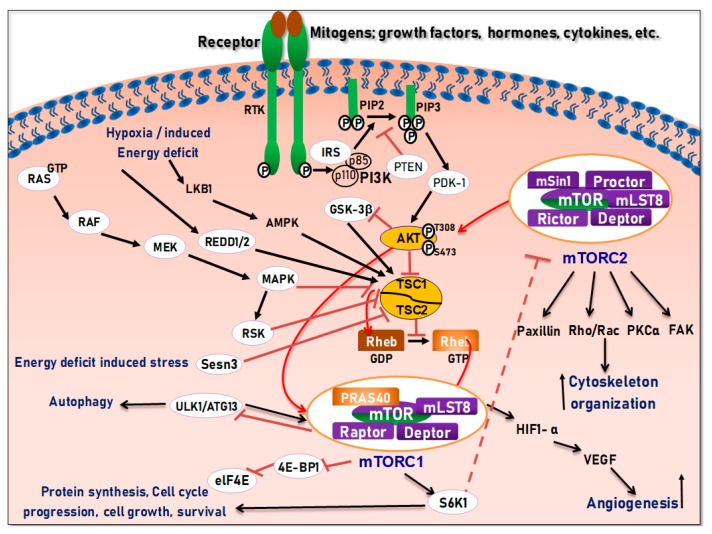
Schematic illustration of the PI3K/Akt/mTOR signaling pathway. Arrows represent activation, whereas bars represent inhibition [50]. Upon receptor activation, insulin receptor substrate (IRS) activates phosphatidylinositol 3-kinase (PI3K), which is in turn phosphorylated to generate phosphatidylinositol [3,4,5]-trisphosphate (PIP3). Phosphatase and tensin homolog (PTEN) can dephosphorylate PIP3 to regulate the pathway activity. AKT is activated through the binding of PIP3 to its amino terminal, pleckstrin homology (PH) domain (stripped), which then promotes the translocation of AKT to the plasma membrane, where the carboxyl terminal T308 is phosphorylated by phosphoinositide-dependent kinase-1 (PDK1), and S473 is phosphorylated by mTORC2. AKT regulates several cellular processes such as survival and cell proliferation, through a variety of downstream proteins like glycogen synthase kinase 3-beta (GSK-3β), Forkhead Box O (FOXO), amid others (not shown). AKT is able to directly phosphorylate and thus inactivates the 40 kDa proline-rich protein (PRAS40), relieving the suppressive regulation on mTORC1 activity. Furthermore, AKT can phosphorylate and inactivate the tuberous sclerosis (TSC) tumor suppressor protein complex that acts as a GTPase-activating protein (GAP) for the RAS homolog enriched in brain (Rheb) small G protein to regulate its activity. Retention of the Rheb-GTP bound form activates mTOR, which is comprised of two main complexes that are associated with diverse proteins such as Raptor, mLST8, PRAS40 and Deptor for complex I (mTORC1), and Rictor, mLST8, Deptor, mSin1 and Protor for complex II (mTORC2). mTORC1 is regulated by a variety of environmental signals mediated via several proteins including REDD1/2 (regulated in development and DNA damage responses 1/2), AMP-activated protein kinase (AMPK), among others. mTORC1 phosphorylates downstream S6K1 (p70S6 Kinase 1) and modulates the eukaryotic initiation factor 4E-binding protein (4E-BP1), which discharges it from hindering eIF4E, and enabling 40S ribosomal subunit to be recruited to mRNAs, leading to the initiation of protein translation. S6K also phosphorylates ribosomal protein S6 that is also involved in translational regulation by the 40S ribosomal subunit. By contrast, the regulation of mTORC2 is still under investigation, but it is known to be regulated by growth factors. mTORC2 phosphorylates distinct groups of proteins, enabling the regulation of actin cytoskeleton and migration via activating protein kinase C α (PKC-α), small GTPases (Rhoa, Rac1 and Cdc42), and focal adhesion proteins, such as focal adhesion kinase (FAK) and paxillin. Essentially, the activation of the RAS-RAF-MEK-ERK-RSK pathway mediated by growth factor is another mechanism of regulated crosstalk with the PI3K/AKT/mTOR signaling pathway.

When talking about mTOR, we have to mention rapamycin. Rapamycin (sirolimus) is an antifungal antibiotic that was first isolated from the bacterial strain *Streptomyces hygroscopicus* NRRL 5491 in 1975 [52,54] in the soil of Rapa Nui Island (Easter Island) from which its name was derived [52]. In 1991, Hall laboratory first discovered target of rapamycin (TOR) in yeast [55,56]. Until mid-1990s, the mammalian counterpart (mTOR) was discovered by Sabatini and colleagues [57]. Rapamycin forms a complex with FK506-binding protein 12 (FKBP-12), and then the rapamycin-FKBP-12 complex binds to the FKBP-rapamycin-binding (FRB) domain of mTOR, inhibiting mTOR function [50]. Thus, mTOR is also termed FKBP-12-rapamycin-associated protein (FRAP), rapamycin and FKBP-12 target (RAFT1), rapamycin target 1 (RAPT 1), or sirolimus effector protein (SEP). mTOR belongs to the PI3K-related protein kinases (PIKKs) family with a C-terminus that shares strong homology to the PI3K catalytic domain (Figure 3). mTOR interacts with several proteins and forms at least two distinctive complexes, namely mTOR complex 1 (mTORC1) and 2 (mTORC2), with distinct kinase activities and cellular functions [46,50,57]. These complexes are large but have different sensitivities to rapamycin as well as different effectors. Both mTORC1 and mTORC2 share the following common components: Catalytic mTOR subunit, mammalian lethal with sec-13 protein8 (mLST8 or GβL), the negative regulator DEP domain containing mTOR-interacting protein (DEPTOR), and the Tti1/Tel2 complex (reviewed in Reference [50]). The mTORC1 discretely comprises the regulatory-associated protein of mTOR (Raptor), and another negative regulator, proline-rich Akt substrate 40 kDa (PRAS40). In addition to the above common components, the mTORC2 additionally contains the rapamycin-insensitive companion of mTOR (Rictor), the mammalian stress-activated MAP kinase-interacting protein 1 (mSin1), and protein observed with Rictor 1 and 2 (Proctor 1/2) (Figure 4) [46,50,57]. Both Raptor and mLST8 are positive regulators of mTORC1′s activity and function, while PRAS40 and DEPTOR are both negative regulators of the mTORC1 [46,52,58]. Raptor serves as a scaffold for recruiting mTORC1 substrates, while mLST8 binds the mTOR kinase domain, and positively regulates its kinase activity. On the other hand, PRAS40 associates with mTOR via raptor to inhibit the activity of mTORC1, while DEPTOR functions as mTOR-interacting protein, to both mTORC1 and mTORC2, as a negative regulator of their activities [50,52].

mTORC1 is sensitive to rapamycin, growth factors, energy (ATP), nutrients (amino acids), oxidative stress, and DNA damage, and regulates cell growth and proliferation by controlling protein/lipid/nucleotide synthesis, and lysosome biogenesis through mediating phosphorylation of S6K1 and eukaryotic initiation factor 4E (eIF4E) binding protein 1 (4E-BP1) (Figure 4) [50]. mTORC2 is only sensitive to prolonged (>24 h) rapamycin exposure in certain cases and growth factors and regulates cell survival and cytoskeletal organization in part by regulating phosphorylation of Akt, serum and glucocorticoid-inducible kinase 1 (SGK1), protein kinase Cα (PKCα) and focal adhesion proteins, as well as the activity of small GTPases [46,57,59]. Though the functions of the mTOR complexes remain to be unveiled, current data indicate that mTOR plays a central role in the regulation of cell growth, proliferation, differentiation, survival, autophagy, and motility, as well as angiogenesis and lymphangiogenesis [46,50,57,59].

## 5. Regulation of the PI3K/Akt/mTOR Pathways in Development and Carcinogenesis

To date, mTORC1 is known to be regulated by multiple pathways (as reviewed below), but how mTORC2 is regulated remains poorly understood. Currently PI3K is the only known upstream modulator of mTORC2, as overexpression of PI3K leads to upregulated mTORC2 activity (Figure 4) [46]. In response to the binding of a growth factor, the corresponding receptor, such as insulin-like growth factor receptor (IGFR), platelet-derived growth factor receptor (PDGFR), or epidermal growth factor receptor (EGFR) on the cell surface, is activated and signals to downstream molecules, leading to the activation of multiple pathways, including the PI3K-Akt and RAS-RAF-mitogen-activated protein kinase kinase (MEK)-extracellular-signal-regulated kinase (ERK)-ribosomal protein S6 kinase (RSK) pathways [57]. The activated PI3K catalyzes phosphatidylinositol [4,5]-bisphosphate (PIP2) to phosphatidylinositol [3,4,5]-triphosphate (PIP3), which is antagonized by the phosphatase and tensin homologue deleted on chromosome 10 (PTEN), a lipid and protein phosphatase (Figure 4). The PIP3 binds to the pleckstrin homology (PH) domain of the serine/threonine kinase, Akt, facilitating Akt docking to the cell membrane, where it is activated via phosphorylation by mTORC2 on S473 and by the phosphoinositide-dependent kinase 1 (PDK1) on T308 [46]. Akt can thus be positively regulated by PI3K, and negatively regulated by PTEN [46,50,57,59]. Therefore, loss of *PTEN* and/or mutations of *PIK3CA* result in constitutive activation of Akt/mTOR, which have been documented in various cancers [52].

Tuberous sclerosis complex 1 (TSC1 or hamartin), TSC2 (or tuberin), and TBC1D7 form a complex, acting as a GTPase-activating protein (GAP) for the Ras homolog enriched in brain (Rheb) GTPase [46,50,57,59]. The GTP-bound form of Rheb interacts with mTORC1 to potently stimulate its kinase activity [46,50,57,59]. Being a Rheb GAP, the TSC1/2 complex negatively regulates mTORC1 by converting an active GTP-bound Rheb into an inactive GDP-bound state [50]. In response to growth factor stimulation, the activated Akt can phosphorylate TSC2 at S939 and T1462, preventing TSC2 from forming a complex with TSC1, so that the active (GTP-bound) Rheb state remains, leading to activation of mTORC1 [46,50,57,59] (Figure 4). Of note, through a TSC1/2-independent manner, Akt can also activate mTORC1 by phosphorylating PRAS40, triggering the dissociation of PRAS40 from raptor [50].

In fact, the TSC1/2 complex can transmit more signals to mTORC1 as well. In response to growth factor stimulation, the activated ERK1/2 and ribosomal S6 kinase 1 (RSK1) can directly phosphorylate TSC2 at S664/540 and at S1798, respectively, inhibiting the TSC1/2 complex and consequently activating mTORC1 [46,50,57,59]. In response to the pro-inflammatory cytokine, tumor necrosis factor-α (TNFα), IκB kinase β (IKKβ) is activated, which can phosphorylate TSC1 at S511/487, causing TSC1/2 inhibition and mTORC1 activation. Furthermore, the canonical Wnt signaling which inhibits glycogen synthase kinase 3β (GSK3-β) can also activate mTORC1 through TSC1/2, considering that GSK3-β is normally responsible for phosphorylation (S1371, S1375, S1379, and S1387) and activation of TSC2 [46,50,57,59].

Furthermore, the AMP-activated protein kinase (AMPK) and/or the regulated in development and DNA damage responses 1 (REDD1), a hypoxia-induced tumor suppressor, can activate the TSCl/2 complex, inhibiting the mTORC1 signaling pathway [46,50,57,59]. The other critically regulated cell growth/survival intracellular signaling pathway is the RAS, which is activated when complexed with GTP, and the conversion of GDP to GTP is inhibited by neurofibromatosis type 1 (NF1), a protein that causes neurofibromatosis type 1 when mutated [57]. As discussed above, downstream of activated RAS are RAF and MEK, which affect the activation of ERK1/2 and RSK1 [46,50,57,59]. The activated ERK1/2 and RSK1 can specifically phosphorylate TSC2, inhibiting the TSC1/2 complex and thereby activating mTORC1 (Figure 4). Consequently, both the PI3K-Akt and the RAS-RAF-MEK-ERK-RSK pathways unite to regulate the mTORC1 signaling [46,50,57,59].

Consequently, the activated mTORC1 further regulates energy metabolism, protein/lipid/nucleotide synthesis, lysosome biogenesis, autophagy, and angiogenesis through S6K1, 4E-BP1, lipin1, activating transcription factor 4 (ATF4), transcription factor EB (TFEB), Unc-51 like autophagy activating kinase 1 (ULK1), hypoxia-inducible factor 1α (HIF-1α), etc. (for details, see review in [53] and [50,57,59]. In particular, activated mTORC1 phosphorylates S6K1 and 4E-BP1, through an interaction between Raptor and a TOR signaling motif in S6K and 4E-BP1. On one hand, activated S6K1 then phosphorylates S6 (40S ribosomal protein S6), thereby improving mRNA translation. This, in turn, further stimulates translation and activates RNA polymerases I and III transcription factors, leading to the synthesis and assembly of ribosomes, tRNAs, and translation factors [57,59]. On the other hand, 4E-BP1 plays an inhibitory role in the initiation of translation by binding and inactivating the eukaryotic translation initiation factor 4E (eIF4E) [50,57,59]. When 4E-BP1 is phosphorylated by mTORC1, it will dissociate from eIF4E. Subsequently, the released eIF4E can bind to eIF4G and eIF4A, forming the eIF4F complex, which binds the 5′ cap of mRNAs and promotes eukaryotic translation initiation [46,57,59].

Unlike mTORC1, little is known vis-à-vis the upstream activators of the mTORC2 pathway. So far, mTORC2 is known to respond to cue from growth factors such as insulin, via direct links to ribosome in a PI3K-dependent fashion [46,50,57]. mTORC2 directly activates Akt via phosphorylation at its hydrophobic motif (S473) and SGK1 (S422), a kinase that control ion transport and growth [46,50,60]. Nonetheless, the loss of mTORC2 does not avert phosphorylation of some Akt targets like TSC2, although on the other hand it completely obliterates the activity of SGK1 [46,50]. Therefore, besides activating mTORC2 via endorsing its association with ribosomes, PI3K, also controls the activation of mTORC1 through the Akt-dependent TSC1/TSC2 inhibition, as described above. It has been proposed that PI3K promotes mTORC2 binding to ribosomes, which directly activates mTORC2; and that mTORC2 activates Akt through phosphorylation at S473 [50,57]. Although mTORC2 is known to be less responsive to rapamycin, prolonged (>24 h) treatment with rapamycin or rapalogs has been reported to inhibit mTORC2 assembly through disruption of the rictor-mTOR complex and consequently inhibiting Akt signaling [46,58]. Of note, there exists a direct phosphorylation of insulin receptor substrate 1 (IRS1) by S6K1, which promotes IRS1 degradation and PI3K/Akt down-regulation [46,50,58]. It has been found that treatment of cancer cells with rapamycin or rapalogs can cause the activation of PI3K/Akt through the S6K1-IRS negative feedback mechanism, reducing the apoptotic potential in cancer cells [46,50,58]. This has become one of the explanations for the unsatisfactory anticancer activity of rapalogs clinically.

In addition, mTORC2 regulates cell actin cytoskeleton and migration via activating protein kinase C α (PKC-α), small GTPases (Rhoa, Rac1, and Cdc42), and focal adhesion proteins, such as focal adhesion kinase (FAK) and paxillin. Therefore, mTORC2 is capable of regulating cell growth, proliferation, survival, and motility [46,58] (Figure 4).

## 6. Cutaneous Cancers Associated with Dysregulation of the PI3K/Akt/mTOR Pathways

Deregulation of the PI3K/Akt/mTOR pathways has been implicated in the pathogenesis of multiple solid human cancers including several skin cancers [46,52]. As a result, this has specifically stimulated the development of specific PI3K, Akt, and mTOR inhibitors for targeted cancer therapy (reviewed in References [46,52,57]). As mentioned above, skin cancers are partly caused by UVA and UVB exposure, which is also associated with the mTOR pathway deregulation [46,50,52,57]. Obligingly, abnormalities due to modifiable (UV) and non-modifiable (genetic) distresses in target genes or proteins of the intracellular networks regulating skin homeostasis, have been shown to result in a gamut of phenotypically varied, and overlapping cutaneous cancers. These cancers are characterized by tissue neoplastic and hyperplastic growth including but not limited to melanoma and non-melanoma skin cancers (basal and squamous cell carcinoma, Merkel cell carcinoma [46,52,57]).

Given that the molecular basis and targets of most skin cancers are well-understood, novel development and effective delivery of chemotherapeutic agents targeting the dysfunction towards maintaining skin tissue homeostasis and integrity are promising therapeutic strategies [46,50,61,62]. In this light, various synthetic small molecule compounds and naturally occurring nutraceuticals have been shown to modulate the activities of the PI3K/Akt/mTOR and may thus serve as novel therapies for these cutaneous cancers [46,50]. Below, we summarize the relevance of the PI3K/Akt/mTOR pathways and the effector molecules in diverse skin cancers and discuss the mechanistic role of several synthetic molecules and dietary phytochemicals in inhibiting these pathways as potential therapeutic approaches.

### 6.1. Role of the PI3K/Akt/mTOR and their Targeting in Melanoma Skin Cancer

Melanoma is a skin cancer derived from malignant transformation of epidermal melanocytes [63,64]. Melanoma is one of the two major forms of skin cancer including non-melanoma skin cancer and is the least common form of skin cancer accounting for only 1% of the total skin cancer incidences, and due to it metastatic potential, is the most aggressive skin malignancy accounting for ~75% of all skin cancer-related deaths worldwide [63,64,65]. The 2018 cancer facts and figures by American Cancer Society reported that about 178,560 cases of melanoma will be diagnosed in the United States. Among those, 87,290 cases will be in situ, or noninvasive and confined to the epidermis, while 91,270 cases will be invasive, or penetrating the epidermis into the dermis. Prognosis of an estimated 9320 people (5990 men and 3330 women) were expected to die of melanoma in 2018 (https://www.skincancer.org/skin-cancer-information/skin-cancer-facts#melanoma). This single fact, in addition to established risk factors for developing melanoma, is imposed by increased socioeconomic burden among melanoma patients with grim 5-years survival depending on metastasis site ranging 12–28% [63,64,66]. Primary benign melanomas are mostly initiated as horizontal lesions with plaque-like appearance in the epidermis, often called the radial growth phase (RGP), which often progresses to the vertical growth phase (VGP), an invasive phase that eventually metastasis to distant organs such as the lung. The transition from RGP to VGP has been reported to be associated with Akt activation, where heightened Akt/mTOR activities has been reported in about 70% of metastatic melanoma [63,67]. Using melanoma models, we recently reported that Akt acts as a molecular switch linked with elevated mTOR, S6K1, angiogenesis, and concomitant production of peroxides, which further nurture the aggressiveness of metastatic melanoma [68].

### 6.2. Targeting PI3K/Akt/mTOR and Associated Pathways with Chemotherapeutics, Biologic Drugs, Natural Products, and Synthetic Derivatives in Melanoma

Activation of the mTOR pathway has been suggested to be strongly associated with the pathogenesis of melanoma [63,64,66,69]. Constitutive activation of mTOR inhibits autophagic cell death and dysregulates the normal cell cycle [70]. Due to advances in knowledge of the molecular genetics of melanoma, novel agents targeting this signal transduction pathway have been developed, including rapalogs (everolimus, deforolimus, and temsirolimus) and mTOR kinase inhibitors [71].

#### 6.2.1. Chemotherapeutic Small Molecules and Biologic Drugs

Several in vitro and in vivo preclinical studies have shown that dual PI3K/mTOR inhibitors have significant inhibitory activities against cell proliferation and activation of Akt, some of which are undergoing clinical trials in patients with selective mTOR mutations (reviewed in Reference [63]). Some synthetic small molecule compounds have been shown to mechanistically target the PI3K/Akt/mTOR and associated RAS/RAF/MEK/ERK or MAPK signaling pathway as promising treatments against metastatic melanoma [63]. Other agents including the BRAF inhibitors dabrafenib and vemurafenib, as well as trametinib, a MEK1/2 inhibitor in metastatic melanoma patients, have yielded extended survival [63]. Moreover, the combination treatment with dabrafenib and trametinib yielded better outcomes in the patients with metastatic melanoma than the individual drug treatment. Despite the rapid development of resistance to these agent treatments, combinatorial approach with additional compounds co-targeting the PI3K/Akt/mTOR (but mostly PI3K), MAPK, and other signal transduction pathways are under investigation at various clinical trials [63].

In addition, mutation of the serine/threonine kinase BRAF is found in almost 50% of the malignant melanoma patients; in more than 90% of the cases BRAF harbors V600E point mutation [72]. Mutation in BRAF activates the MAPK pathway, which is involved in cancer cell survival and proliferation. Inhibition of BRAF is also a promising approach in treating malignant melanoma. A number of small molecules have been introduced by far to inhibit BRAF, some of which are approved by the FDA for targeting BRAF mutated malignant melanoma. However, resistance to such molecules is quite common leading to therapeutic failure. A number of mechanisms contributing to escape the BRAF inhibition have been reported in several studies. One of the studies reported the involvement of hepatocyte growth factor (HGF) in acquisition of resistance against BRAF inhibitors through upregulation of c-MET and GAB1, leading to activation of the MAPK pathway [73]. Abnormal expression of long non-coding RNAs (lncRNAs) has also been implicated in the metastatic growth of cells in many cancer types. Activation of c-MET lncRNAs KCNQ1OT1 [74], or downregulation of tumor suppressor microRNA MiR-22 by MALAT1 [75] and miR-152–3p by HOTAIR [76] was also found to increase metastatic growth of melanoma cells [74]. Treating the melanoma cells with combination of BRAF inhibitor Vemurafenib and c-MET inhibitor AMG 337 [73] or siRNA exhibited therapeutic benefits in BRAF mutant malignant melanoma. Hersey et al. reported that the combination of small chemotherapeutic molecules and targeted biological therapies was clinically beneficial in distant metastatic disease [71].

Rapamycin, a specific mTORC1 inhibitor inhibits the cell growth and proliferation as shown in several melanoma cell lines [77,78]. Two other rapamycin analogs, everolimus and temsirolimus, also showed promising results in preclinical studies, inducing cytostatic tumor growth inhibition and decreasing angiogenic capillary perfusion. However, in a phase II clinical study, everolimus failed to demonstrate adequate efficacy in treating patients with metastatic melanoma; but its antiangiogenic role suggested potential utilization in combination therapy [79]. Another mTOR inhibitor, temsirolimus, in combination with chemotherapeutic agent temozolomide, exhibited significant decrease in tumor growth and increased apoptotic death in melanoma cells that showed resistance to BRAF inhibitor vemurafenib [80]. In a phase I clinical study, the combination of temsirolimus and an autophagy inhibitor hydroxychloroquine, accelerated the cell death in melanoma [81].

In a phase 2 trial from the Sarah Cannon Oncology Research Consortium, Hainsworth et al., reported that the combination of Bevacizumab and everolimus was tolerable and showed moderate activity in the treatment of patients with metastatic melanoma [82]. This is an indication that further investigation of compounds with such mechanisms of action, even in combination with inhibitors of secondary signaling pathways, are promising research areas [82]. VS-5584 is a low-molecular weight compound and a novel potent and highly selective dual PI3K and mTOR inhibitor that is well tolerated in animal models with good pharmacokinetic properties [67,83]. Since melanoma is highly resistant to conventional chemotherapeutics, by investigating the in vitro and in vivo anti-melanoma activity of VS-5584, Shao et al. reported a significant and simultaneous blockade of activated components of Akt/mTOR pathway as well as the downregulation of cyclin D1 expression in melanoma, indicating its utility as a potent PI3K/mTOR dual inhibitor [67,84,85,86]. Moreover, the activity of orally administered VS-5584 suppressed the growth of A375 melanoma xenograft in nude mice. Co-administration of ABT-737 (Bcl-2 inhibitor) with VS-5584 further enhanced the suppressive effects, suggesting a rationale for the clinical assessment of VS-5584 in melanoma patients as well as the development of ABT-737 and other target inhibitors in adjuvants settings [67].

SKLB-M8 is a derivative of millepachine (MIL, a novel chalcone with a 2,2-dimethylbenzopyran motif derived from Millettia pachycarpa Benth (*Leguminosae*), and a flavonoid-rich traditional Chinese medicine). The modified derivative of MIL, (*E*)-3-(3-amino-4-methoxyphenyl)-1-(5-methoxy-2,2-dimethyl-2*H*-chromen-8-yl) prop-2-en-1-one hydrochloride (SKLB-M8) [87,88] has been reported to have antitumor and especially anti-melanoma activity. Wang et al. reported that SKLB-M8 treatment inhibited the proliferation and the expression of cdc2, induced G2/M arrest, and elicited apoptosis via the down-regulation of activated Akt/mTOR signaling pathway in melanoma models as well as inhibited angiogenesis associated with inhibition of ERK1/2 phosphorylation (Table 1) [87,88].

Due to the superior benefit of multi-target strategy over single target approach, Oudart et al. employed the Type XIX collagen NC1 domain associated with basement membranes [NC1 (XIX)], a 19-amino acid peptide localized at the C-terminal end of the α1 (XIX) chain in multi-target identification in an anti-melanoma approach [89] (see Table 1). Using the tumor NC1 domain of collagen Type XIX [C1(XIX)], Oudart et al. were able to target the αvβ3 integrin interaction and demonstrated the inhibition of migration, invasion, and PI3K/Akt/mTOR and FAK pathways in melanoma cells and preclinical melanoma model [89,90,91].

Itraconazole is another FDA-approved azole compound that belongs to the antifungal drug family, which has been repurposed for the treatment of various cancers including melanoma [110,111]. Report by Liang et al., demonstrated an anti-melanoma effect of itraconazole and indicated the molecular mechanism to include the inhibition of the PI3K/mTOR, and Hedgehog/Wnt pathways [110].

Increasing evidence has identified the aberrant expression of miRNAs (microRNAs) in melanomagenesis including Uveal melanoma (UM) [114,115]. Jiang and Liu reported that miR-25 target the RNA-binding motif protein 47 (RBM47) and activated the PI3K/Akt/mTOR signaling pathway in melanoma cells [116]. Meng et al. recently demonstrated clinically that miR-138 is significantly upregulated in malignant melanoma patients by regulating the PI3K/Akt/mTOR autophagy pathway via PDK1 dependent expression [117]. Moreover, a study by Li et al. also reported that by targeting PIK3R3/AKT3, miR-224-5p suppressed proliferation, migration, and invasion in uveal melanoma (UM) cells, representing a therapeutic and diagnosis target for patients with UM [115]. Furthermore, Micevic et al. reported that the loss of the overexpressed DNA methyltransferase (DNMT3B) that plays a pro-tumorigenic role in human melanoma resulted in a dramatic suppression of melanoma formation in the Braf/Pten mouse melanoma model [118]. This loss also resulted in hypomethylation of the promoter of miR-196b and a subsequent increase in the expression of miR-196b, which directly targets Rictor (mTORC2 component) to inhibit the activation of mTORC2, which is dire for formation and growth of melanoma. Thus, this study establishes DNMT3B as a regulator of melanoma development via its influence on mTORC2 signaling and suggests a new therapeutic target in melanoma [118].

Moreover, since aggressive and highly metastatic cutaneous melanoma involves the overexpression of Rictor, the major regulator of Akt phosphorylation, the effect of Rictor inhibition in melanoma models with specific accent on liver metastasis has been investigated. Schmidt et al. reported for the first time that mTORC2/Rictor play a critical role in melanoma liver metastasis via the interactions between cancer cells and cancer-associated hepatic stellate cells (HSC); inhibition of mTORC2/Rictor led to significant inhibition of Akt phosphorylation and motility of the cancer cells [119]. Additionally, Damsky et al. reported that mTORC1 activation blocked BrafV600E-induced growth arrest but was insufficient for the arrest of melanoma development, concluding that activation of both mTORC1/2 is required for Braf-induced melanomagenesis [120].

Rapamycin, its analogs and other protein kinase inhibitors have been investigated for targeting the main mTOR hub in melanoma cells. Ciołczyk-Wierzbicka et al. investigated the role of rapamycin (mTOR), everolimus (mTOR), U0126 (ERK1/2), LY294002 (PI3K), CHIR-99021 (GSK-3β) and others in human VGP (WM793) and metastatic (Lu1205) melanoma cells and observed their antiproliferative effects, in view of the crucial role of the PI3K/Akt/mTOR and ERK1/2 signaling pathways in melanoma progression [121] (Table 1). Temsirolimus (Torisel) targets multiple hallmarks of cancer to impede melanoma growth in vivo. An earlier study has shown that everolimus (RAD-001), an orally active rapamycin analog, may be potentially beneficial to treatment of metastatic melanoma (NCCTG-N0377, Alliance). However, a phase II trial reported that the single treatment with everolimus did not exhibit sufficient anticancer activity, suggesting that a combination with other drugs be considered in future clinical studies [79]. In a recent phase II study of everolimus against advanced melanoma patients with mTOR mutations, Si et al. reported that mTOR inhibitors had restricted activity in non-selected melanoma patient population, whereas a significant percentage of mTOR mutation melanoma patients responded better [92]. The study suggested that it may be possible for future prospective studies to identify suitable patients that would respond to mTOR inhibitors treatment (Clinical trial information: NCT01960829). Rao et al. recently conducted a two-stage Phase II multi-institutional trial evaluating everolimus (RAD-001) in the treatment of patients with metastatic melanoma by assessing progression free survival with baseline derived from historical controls (DOI: 10.1200/jco.2006.24.18_suppl.8043). They observed from interim analysis of 20 patients that RAD-001 was well tolerated with sufficient anti-metastatic melanoma activity. This result encouraged them to have open enrollment to the second stage of this trial.

PBISe (Se,Se′-1,4-phenylenebis(1,2-ethanediyl)bis-isoselenourea) is a selenium containing isosteric analog of the iNOS inhibitor PBIT [*S,S*′-1,4-phenylenebis(1,2-ethanediyl)bis-isothiourea], which has been utilized as a small molecule to treat systemic metastasis of melanoma [122]. Recently, Chung et al. showed that topical application of PBISe prevented cutaneous melanocytic lesion or melanoma development at 70–80% in reconstructed melanoma skin model and approximately 50% in melanoma tumor xenograft in mice via the downregulation of the activated Akt signaling, and a concomitant activation of ERK1/2 pathway. The data suggest that PBISe treatment concurrently targeting both pathways has potential to prevent cutaneous metastatic melanoma development in skin [102].

Perifosine, an alkylphosphocholine analog and Akt inhibitor, failed to demonstrate a good efficacy in a phase II clinical trial with various side effects and biochemical toxicity, suggesting no further development of this single compound for recurrent melanoma in human [105,123].

PI-103, a kinase inhibitor targeting Class I PI3K and both mTORC1 and mTORC2, has been shown to exhibit only modest anti-proliferative, cytotoxic effect in vitro and in vivo murine model as a single agent. However, Werzowa et al. showed that the combination of PI-103 and rapamycin in vitro (in melanoma cells) and in vivo (in a melanoma mouse model), synergistically induced apoptosis and suppressed Akt/S6 protein phosphorylation with superior efficacy against malignant melanoma [104].

#### 6.2.2. Natural Plant-Derived Extracts, and Phytochemicals and their Synthetic Derivatives

Additionally, natural dietary phytochemicals have been proven to be effective in many cancer types including melanoma, and have generated encouraging anti-proliferation, anti-invasive, and anti-metastatic effects, which are often associated with their ability to target PI3K/Akt/mTOR and other signaling pathways involved in melanoma carcinogenesis (melanomagenesis). Since several of these natural phytochemicals have lower toxicities and side-effects at physiological attainable doses, exploration of their utility in a single basis or in combination or in adjuvant settings to this plethora of known anticancer targets are laudable therapeutic opportunity. Outlined below are a non-exhaustive list of examples that have proven useful in melanoma.

Acacetin (5,7-dihydroxy-4′-methoxyflavone) is a natural flavonoid derived from *Robinia p seudoacacia*, also termed black locust, with well documented antioxidant, anti-inflammatory, and anticancer properties [124,125]. Its anticancer effect has been associated with modulation of the PI3K/Akt/IKK and the MLK3/MKK3/6 and p38 MAP kinase pathways (reviewed in Reference [124]). Using cell-free, biophysical, computational, cell-based, and in vivo melanoma xenograft models, Jung et al. reported that acacetin inhibited PI3K activity, suppressed Akt phosphorylation and significantly regressed SK-MEL-28 melanoma tumor growth in vivo, suggesting an anti-melanoma agent [124].

Capsaicin (trans-8-methyl-*N*-vanillyl-6-nonenamide) is an active compound found in chili pepper that has been shown to inhibit migration and angiogenesis of B16-F10 melanoma cells in vitro; the mechanism of action was via the inhibition of the PI3K/Akt/Rac1 signal pathway [126].

Evodiamine is a major natural alkaloid component of *Evodiae fructus*. Evodiamine treatment of human melanoma A375-S2 induced cell death that was mediated through PI3K/Akt/caspase and Fas-L/NF-κB signaling pathways and this effect was synergized upon treatment with ubiquitin-proteasome inhibition [127]. 

Isoliquiritigenin (ISL) is a natural flavonoid with known ability to reprogram cancer cells with in vitro and in vivo anticancer activity. ISL treatment significantly inhibited A375 melanoma cell proliferation, induced G2/M cell cycle arrest, and up-regulated terminal melanocyte differentiation indicators, and significantly decreased melanoma cachexia through decreasing the protein expression levels of activated mTORC2-Akt-GSK3β signaling pathway components. Combined-treatment of ISL and Ku-0063794 (mTOR-specific inhibitor) synergistically inhibited proliferation, and increased melanocyte differentiation markers than ISL alone treatment [128].

Melittin, an amphiphilic small peptide containing 26 amino acid residues, is the major active ingredient from bee venom (BV) with known anti-inflammatory, antibacterial and anticancer activities. It has been demonstrated that BV and melittin have antimelanoma effects with mechanisms associated with the suppression of the activation of the PI3K/Akt/mTOR and MAPK signaling pathways. Moreover, the combination of melittin with temozolomide (TMZ; chemotherapeutic agent) significantly inhibited growth and invasion of melanoma cells compared to the individual single agents, strongly suggesting that melittin could be a potential anti-melanoma agent [129].

Panduratin A, a phytochemical isolated form the rhizome of *Boesenbergia rotunda* was reported in many studies to show effectiveness in several cancer types. In a recent study it has been shown to induce autophagic cell death in melanoma cells that are resistant to apoptosis-inducing chemotherapeutics. Induction of autophagy by panduratin A was found to be mediated by suppression of the mTOR signaling pathway [130].

Another multicomponent Chinese herbal preparation Compound Muniziqi granule (MNZQ), which contains 13 medicinal plants, has been used traditionally for the treatment of endocrine disorder-induced acne, chloasma, dysmenorrhea, menopausal syndrome, and melanoma. One of the components of the MNZQ is *Peganum harmala* plant seed extract, which contains β-carboline alkaloid, harmine. Harmine has been found to induce apoptosis and autophagic cell death in mouse B16 melanoma cells. The autophagy induced was due to the inhibition of multiple signaling pathways which include the Akt/mTOR and ERK1/2 signaling pathways [131].

Sinomenine (7,8-didehydro-4-hydroxy-3,7-dimethoxy-17-methylmorphinane-6-one), the main active component of the medicinal plant *Sinomenium acutum*, was also shown to induce apoptosis and decrease proliferation in B16 melanoma cells and mouse tumor xenografts. The apoptotic and antiproliferative effect of sinomenine is due to induction of autophagy by inhibiting the PI3K/Akt/mTOR signaling pathway [132].

Studies by Espona-Fiedler et al. demonstrated that the anti-melanoma effect of two small molecules members of the prodiginines family, prodigiosin and obatoclax, is related to inhibition of both mTORC1 and mTORC2. Of note, prodigiosin and obatoclax inhibited Akt phosphorylation at S473, despite no effect on T308 [133].

Fisetin (3,7,3′,4′-tetrahydroxyflavone), a dietary bioactive flavanol abundantly found in pigmented fruits and vegetables including apples, cucumbers, onions, persimmons, and strawberries, has been reported to possess pleiotropic effects in diverse human diseases including cancer [134,135,136]. Fisetin has been investigated for treatment cutaneous cancer, particularly melanoma, having constitutive activation of the Akt/mTOR signaling, due to mutations of PTEN, PIK3CA, or TSC1/2 [77,137,138]. Syed et al. reported that fisetin targeted several key melanomagenesis markers by diverse mechanisms, including inhibition of the Akt/RSK/mTOR/S6K axis, suggesting that fisetin is a potent anti-melanoma agent [68,139,140]. Pal et al. in a study of the effect of fisetin alone and in combination therapy with sorafenib, demonstrated that fisetin enhanced sorafenib-induced apoptosis and abrogated tumor growth in athymic nude mice xenografted with BRAF-mutated melanoma cells through the inhibition of the expressions of activated components of PI3K and MAPK pathways [141]. The data suggest that simultaneous inhibition of both PI3K and MAPK pathways by the combination of fisetin and sorafenib may be a better anti-melanoma therapeutic option.

Curcumin (diferuloylmethane), a polyphenolic active component of turmeric, derived from the rhizome of the plant *Curcuma longa*, has shown a wide variety of health benefits including anti-inflammatory, antioxidant and pro-apoptotic effects in diverse cancers by modulating multiple signal transduction pathways. By treating human melanoma cells with curcumin, Zhao et al., reported the induction of autophagy, and concomitant inhibition of proliferation and invasion via the suppression of activated components of the Akt/mTOR signaling pathway [142]. Another study by Rozzo et al., showed that an analog of curcumin (D6) significantly inhibited proliferation and induced apoptosis in melanoma cells involving the down-regulation of the PI3K/Akt and NF-κB pathway [143].

Resveratrol (*trans*-3,5,4′-trihydroxystilbene), a natural bioactive phenolic compound commonly found in pigmented fruits such as cranberries, grapes, and peanuts, has been shown to exhibit multifaceted biological and health beneficial effects by targeting multiple disease molecular markers [144,145]. Wang et al. showed that resveratrol treatment of B16 melanoma cells resulted in induction of autophagy in a mechanism involving the accumulation of ceramide and inhibition of Akt/mTOR pathway, suggesting a potential in treating melanoma [144,146]. Moreover, Bhattacharya et al. demonstrated that treatment with resveratrol reduced cell migration and invasion, and inactivated Akt/mTOR effectors in malignant melanoma and fibroblast cell lines [46,145].

Honokiol, a natural phenolic compound used for a long time in Chinese and Japanese traditional medicine, has recently been shown as a promising anticancer agent. Kaushik et al. reported that honokiol treatment resulted in the induction of cytotoxicity and cytostatic effects by inhibiting the Akt/mTOR and Notch signaling pathways in malignant melanoma cancer cells [63,147].

Epigallocatechin-3-gallate (EGCG), the most abundant catechin found in green tea (*Camellia sinensis*), has shown several health beneficial effects including anticancer activity (Table 2). In particular, EGCG possesses in vitro and in vivo pharmacological effects of EGCG on the migration and/or metastasis and on the management of melanoma, by inhibiting the PI3K and several signaling pathways like Reference [148].

Nexrutine^R^, derived from *Phellodendron amurense*, is an inducer of oxidative stress, inhibiting antioxidant response (Table 2). Since melanoma cells exhibit heightened oxidative stress phenotype associated with increased protein damage, oxidized glutathione, reactive oxygen species (ROS), and KEAP1/NRF2 pathway activity, compared with normal melanocytes contributing to hyperactive proliferation and increased survival, agents targeting these can curtail the disease [149]. Hambright et al. reported Nexrutine^R^ treatment augmented the constitutively elevated oxidative stress markers, reduced proliferation, survival, and colony formation in melanoma cells, which was associated with selective inhibition of activated components of the PI3K/Akt/mTOR pathway [149,150].

### 6.3. Targeting PI3K/Akt/mTOR for Treatment of Basal Cell Carcinoma

Keratinocyte carcinoma (KC) comprises basal cell carcinoma (BCC) and cutaneous squamous cell carcinoma (cSCC), which constitute the main forms of non-melanoma skin cancers (NMSC) [152,153]. BCC is the most common type of non-melanoma skin cancer among Caucasians or ethnic groups having blue or green eyes, blond or red hair, and light-colored skin exposed to the sunlight for prolonged periods of time [154,155,156]. BCC arises in the basal epidermal cell layers and constitutes up to 80% of skin cancers and nearly a third of all cancers diagnosed in the United States [157,158,159] with an incidence rate of up to 5% per year generating a total cost of about $400 million/year [160]. BCC is usually not life threatening, but if left untreated it can cause loss of function, and disfiguration [161,162,163,164]. The morphological features of BCC include the presence of a group of tumors in the dermal layer of the skin that are composed of cells having cellular components similar to undifferentiated basal epidermal cells. An important feature of BCC is the palisade arrangement of epidermal cells in the tumor periphery that separates the tumor from the surrounding stroma. These cells often give the tumor nodular shape or form a band or string surrounding it. Compared to their normal counterparts, the tumor cells have less cytoplasm and chromatin-rich nucleus, which renders more frequent mitotic division but at the same time apoptotic cell death accounts for slow progression of the tumor [165] discussed above, the primary risk factor for BCC being the direct exposure to the sunlight (UV-A and UV-B radiation) and depends on the rate, extent, and duration of exposure to UV irradiation. Other risk factors for BCC include immunosuppression, trauma, arsenic poisoning and other skin disorders such as Gorlin-Goltz syndrome or xeroderma pigmentosum [166,167]. The clinical presentation of BCC may appear in different morphological patterns such as nodular or cystic, superficial, infiltering, and sclerotic or pigmented, which differ in their site of occurrence as well. The nodular or cystic BCC usually occurs as solitary, shiny, red nodules on the face whereas the superficial BCC tends to occur in the trunk. Infiltering BCC is the most aggressive type of tumor often with less defined border [155,165]. Upregulation of the Hedgehog signaling has been found to be the primary mechanism of BCC development, though other non-canonical pathways such as WNT, NOTCH, p53 as well as the P13K/Akt/mTOR pathways have been implicated in the pathogenesis of BCC.

Po-Lin So et al. demonstrated that even a brief inhibition of the PI3K/Akt/mTOR pathway can sustain the inhibition of BCC carcinogenesis long after treatment is completed [106]. This suggested that short-term exposure of BCCs to PI3K inhibitors can result in chemoprevention or chemotherapy, and consequently, evades toxicity and side effects that often result from chronic treatment with other antitumor agents such as tazarotene [106]. Everolimus (inhibitor of mTORC1) shows antiproliferative activity against several types of neoplasia, particularly against BCC. An oral daily dose of 1.5–3 mg everolimus has demonstrated significant improvement in BCC patients with partial or complete recession of the disease [93]. Since the crosstalk among the different signaling pathways such as p53, WNT, Hedgehog, NOTCH along with the P13K/Akt/mTOR pathway exists in BCC, resistance to a particular pathway inhibitor is common. In this scenario, combination treatment with inhibitors of different signaling pathways has been proven beneficial. The PI3K inhibitor, buparlisib, in combination with the smoothened (SMO) inhibitor, erismodegib, in Hedgehog signaling pathway is currently under investigation for determining the efficacy of the treatment in BCC [166]. Furthermore, there is a considerable crosstalk among these pathways creating an intricate network of molecules which confers resistance to the drugs targeting a particular signaling [93]. GANT61 (inhibitor of GLI) in the Hedgehog pathway has been tried to establish an efficient mode of targeting BCC. However, the crosstalk between the PI3K and Hedgehog signaling has evolved the resistance against the inhibitors of Hedgehog signaling. Combination of PI3K/mTOR inhibitor, PI103 and GANT61, has been shown to synergistically overcome the resistance in a rhabdomyosarcoma model [166]. Another PI3K inhibitor buparlisib is under investigation to determine the effectiveness of the SMO inhibitor erismodegib in BCC [166]. Pharmacological doses of retinoids are reported to be effective in BCC carcinogenesis in human. The retinoid, tazarotene, has been shown to inhibit murine BCC through inhibition of IGFR/PI3K/Akt/mTOR signaling pathway [106,107].

Drug repurposing has led to the identification of novel anticancer drugs through the discovery of new pharmacological effect of the existing drugs with established therapeutic activity. Itraconazole was discovered as an antifungal agent in the 1980s, which in recent years has been discovered to have anticancer activity through inhibition of different signaling pathways such as Akt/mTOR, Hedgehog signaling, and Wnt/β-catenin signaling. Itraconazole has been found to inhibit the mTOR signaling by binding to the voltage sensitive anion channel in mitochondria and interfering with ATP production. Itraconazole showed a promising result in BCC treatment in recent clinical trials and is also being used in ongoing clinical trials for BCC [168]. Pharmacological doses of retinoids are reported to be effective in BCC carcinogenesis in human. The retinoic acid receptors (RAR) α, β, and γ in the endodermal layer of the skin bind the retinoic acid derived from endogenous conversion of retinoid to retinoic acid and retard BCC carcinogenesis. A typical example is tazarotene, a prodrug, when converted to tazarotenic acid, can potently bind and activate RAR β and γ receptors, thus exerting the anticancer effect [106,107].

### 6.4. Targeting PI3K/Akt/mTOR for Treatment of Cutaneous Squamous Cell Carcinoma

Cutaneous squamous cell carcinoma (cSCC) is the second most common form of non-melanoma skin cancer globally after BCC, accounting for almost 20% of NMSCs [169], and clinical presentation including but not limited to hyperkeratotic plaque, formation of nodular mass or ulceration on the skin, which may be associated with pain, pruritus, or bleeding [170]. Actinic Keratosis AK and Bowen’s disease [171] are two premalignant forms of cSCC, which, if not treated well, result in malignant transformation. Although 95% of the cSCC are curable by surgical means, it is estimated that 20% of the skin cancer deaths are instigated by cSCC [172]. The cSCC can be metastasized to the surrounding dermis layer and to the local lymph nodes. Approximately 5% of the patients have been found to develop metastasis in the lymph nodes [173]. Exposure to ultraviolet radiation from the sunlight is the major cause of cSCC development. Other causes include occupational exposure to ionizing radiation and radiotherapy for the treatment of other skin conditions such as psoriasis. Immunosuppressive treatment followed by organ transplantation has also been found to be implicated with development of cSCC. Prolonged administration of immunosuppressant azathioprine was found to increase the risk of cSCC in patients after lung transplantation [174].

Overexpression of EGFR has been reported in cSCC, so targeting EGFR has been a promising therapeutic approach in treating this type of cancer. In a phase II clinical study EGFR tyrosine kinase inhibitor gefitinib showed a promising result in patients with aggressive cSCC of the head and neck [175]. Furthermore, cSCC has been reported to exhibit a higher level of mTOR activity compared to other non-melanoma skin cancers particularly BCC [176,177]. Although cSCC shows more aggressive behavior than BCC, interestingly it shows better response to mTOR inhibitors due to higher mTOR level in these cell types [178]. Elevated mTOR level was also observed in cSCC, compared to its premalignant forms AK and BD. Increased expression of cyclin-dependent kinase 2 (CDK2) was also observed in this type of cancer, suggesting its correlation with the Akt/mTOR signaling. This may act as another potential therapeutic target for treating cSCC in addition to targeting mTOR [179]. Since rapalogs (sirolimus, everolimus, etc.) have immunosuppressive effects and at the same time also possess antiproliferative activity, these mTOR inhibitors can be promising agents in treating patients with malignancies following organ transplantation. Rapalogs have been reported to be effective in posttransplant skin malignancies, especially in cSCCs [180]. In a recent study, a novel orally bioactive PI3K/mTOR dual inhibitor, LY3023414, has been found to inhibit human cSCC and is currently under phase I/II clinical trials for the treatment of patients with cSCC [112]. Einspahr et al. utilized reverse phase protein microarray analysis to study independent tissue sets of isolated and enriched epithelial cells squamous cell carcinoma (SCC) compared with actinic keratosis (AK) obtained by laser capture microdissection, and observed aberrant activation of MEK-ERK, EGFR, and mTOR pathways [181]. In another study, Chen et al. reported frequent constitutive activation of the Akt/mTOR pathway components in mostly malignant epidermal tumors, which also highly correlated with CDK2 expression, suggesting that this pathway induces the malignant transition through CDK2 in epidermal tumors [179].

The PI3K/Akt/mTOR signaling pathway has been implicated in the development of resistance to EGFR inhibitors in head and neck SCC. Therefore, everolimus (mTORC1 inhibitor) was combined with erlotinib (EGFR inhibitor) in a phase II clinical trial to treat head and neck SCC. Unfortunately, this combination treatment did not show any clinically significant benefits to the patients with metastatic cancer [182].

Erufosine (erucylphospho-*N*, *N,N*-trimethylpropylammonium), an alkylphosphocholine, showed promising anticancer effects in oral squamous cell carcinoma. The induction of apoptotic and autophagic cell death and the antiproliferative effect of erufosine resulted from its downregulatory effect on the mTOR signaling cascade [94].

Several small molecules have been developed to target mTOR signaling to treat cSCC. Some of these molecules have shown promising results in ex vivo experiments. GDC-0084, a novel small molecule showed potent inhibitory effect on both mTORC1 and mTORC2. It exhibited antiproliferative and cytotoxic effects on several established and primary cSCC cell lines. A clinical study in human subjects showed the good safety and tolerability as well as the complete shutdown of the PI3K-Akt-mTOR signaling cascade, presenting GDC-0084 as a potential therapeutic agent in treating cSCC [95]. Another PI3K/mTOR dual inhibitor, LY3023414, also showed potent cytotoxic activity in several cSCC cell lines as well as the in vivo tumor xenograft models. The high aqueous solubility and oral bioavailability positioned LY3023414 as a potential chemotherapeutic agent for treatment of cSCC. This small molecule is currently undergoing phase I and II clinical trials [112].

### 6.5. Targeting PI3K/Akt/mTOR for Treatment of Merkel Cell Carcinoma

Merkel cell carcinoma (MCC), first discovered by Toker in 1972, is a devastating nonmelanoma skin cancer of neuroendocrine origin, which contains neurosecretory granules with an increasing/grim prevalence of 1500 cases per year in the US alone. A five-year epidemiologic data revealed in Australia and other parts of the world that there is an annual increase of 8% in an age adapted incidence of MCC compared to only 3% corresponding increases in cutaneous melanoma. About 50% of MCC patients progressed to metastatic disease with an associated 46% mortality rate, which thus far exceeds that of melanoma [109]. In spite of this increase, approximately 11-fold greater cases of MCC have been reported in the patients with AIDS. The electron-dense neuroendocrine granule containing Merkel cells originate from epidermal stem cells [183]. MCC most commonly occurs in the sun-exposed areas of the body especially the head and neck, and the pathogenesis remains incompletely understood.

In 2008, Merkel cell polyomavirus (MCV) was discovered as a causative agent of MCC, suggesting that integration of the viral genetic material to the cell is responsible for the virus induced pathogenesis, and the molecular mechanism associated with the disease pathogenesis has been reported in diverse literatures, which involves p53, PTEN, Ras/MAPK, and PI3K/Akt. A recent report suggested the involvement of activated Akt/mTOR and its downstream effector molecules p-4E-BP1 (S65) and p-S6K in MCC cells [109], and a positive correlation between MCV specific T cell antigen and the translation initiation factor 4E-BP1 was testified to activate Akt/mTOR signaling in MCV positive tumor [184]. It was also shown that constitutive activation of 4E-BP1 that preserved from being phosphorylated upset the MCV specific T cell transformation activity, suggesting that 4E-BP1 inhibition (phosphorylation) is required for MCV transformation [109,184]. More studies have reported that Akt is hyper-phosphorylated in MCC regardless of the presence of MCV [109,184].

There are no real treatments for the MCC, even though the first-generation inhibitors of mTOR including rapamycin and other rapalogs are currently being investigated in different cancer types including some skin malignancies. Diminished efficacy of rapalogs due to feedback activation of Akt has instigated the development of the second-generation mTOR inhibitors that can target both mTORC1 and mTORC2. For instance, MLN0128, a compound that inhibits both mTORC1 and mTORC2, was able to potently suppress the growth of MCC both in vitro culture and in vivo in mouse xenograft models. MLN0128 is now at the verge of a dose escalation protocol in phase I clinical trial and undergoing a phase II clinical trial for the treatment of MCC [98,99].

WYE-354 is an allosteric mTOR inhibitor that has been reported to increase autophagy in primary human MCC cell line with over two-fold more efficacious than Ku-006394 in 24 h treatment regimen [109].

Another small molecular target of PI3K/Akt/mTOR pathway is NVP-BEZ235, which inhibits the activity of both PI3K and mTOR kinase by interacting with the ATP-binding site. It is an imidazoquinoline derivative with significant oral bioavailability. In different preclinical trials NVP-BEZ235 has been reported to be effective against osteosarcoma, glioblastoma, breast, prostate, and pancreatic cancer and is now in phase I clinical trial for solid tumors. This molecule has been found to inhibit proliferation and induce cell cycle arrest of MCC cells in culture. The mechanism of NVP-BEZ235 action against cancer cell proliferation was found to be associated with dual inhibition of PI3K/mTOR [101].

### 6.6. Targeting PI3K/Akt/mTOR for Treatment of Tuberous Sclerosis

Tuberous sclerosis complex (TSC) is an inconsistently expressed, mostly autosomal dominantly inherited neurocutaneous syndrome/disorder that affects the skin, brain, kidneys, eyes, and other organ systems known to affect persons of several races, including those from sub-Saharan Africa or of black, African, ancestry [185,186]. TSC exhibits a wide spectrum of clinical sequelae, pathologically characterized by benign, non-invasive, tumor-like lesions or organs hamartomas). These typically occur in multiple organs including the central nervous system, kidneys, lungs, and skin (observed as hypomelanotic macules, confetti skin lesions, facial angiofibromas, shagreen patches, fibrous cephalic plaques, or ungual fibromas) [187,188,189]. At molecular levels, mutations of TSC genes modulate proliferation and differentiation of cells, causing the hamartomas, tumors, or altered neuronal polarity. It has been reported that most persons affected by TSC defects display distinctive phenotypes associated to attacks, including childhood spasms with autism related to variable intellectual disability [188,190]. It is commonly estimated that TSC is a rare condition with an average frequency of 1:6000 live births, and the prevalence ranges between 1:14,000 and 1:25,000 [189,191], and mutations in one of the two tumor suppressor genes, TSC1 (encoding hamartin) or TSC2 (encoding tuberin) occurs in more than 85% of TSC cases [192]. Under abnormal conditions, hamartin and tuberin are activated to avoid substrate usage through the biosynthesis courses facilitated by the mTORC1. Importantly, mutation of either TSC1 (on chromosome 9) or TSC2 (on chromosome 16) in patients with TSC leads to dysfunction of hamartin or tuberin. This causes the activation of mTOR signaling, leading to anomalies resulting from downstream kinase signaling cascade with deregulated cell cycle progression, transcription, translation, and metabolic control [193,194]. The stratification of mosaic TSC into subtypes corresponding to disease prognosis and severity, identified phenotypic distinctions between mosaic forms of TSC. They reported mosaic patients with; asymmetrically distributed facial angiofibromas, bilaterally symmetric facial angiofibromas, and germline TSC, involving both cutaneous and internal organs [186,195,196]. Some neonates are characterized by cardiac failure due to intracardiac rhabdomyomas, and often can show an age-dependent increase in the likelihood of developing renal angiomyolipomas. Central nervous system tumors are the principal basis of morbidity and mortality, while renal disease comes second as leading cause of early death due to TSC [189]. The pathophysiology and the clinical diagnostic and therapeutic management of TSC has recently been reviewed [191]. A high morbidity and mortality due to pulmonary involvement occurs predominantly in women. As explained above, both proteins form a functional complex that modulates the mTOR pathway, as such drugs that suppress mTOR are currently employed to treat TSC-related tumors and are being investigated as potential agents to alleviate other complications associated with TSC. Treatment of TSC patients possess severe burdens time wise and costly healthcare for both the patients, their families, as well as the healthcare system with no acceptable standard treatment approach that are clinically and economically effective [191]. A consensus was reached that a multidisciplinary strategy is required for ideal care of TSC patients [188]. Current treatment and care options are conservative including surgery, pharmacologic treatment with mTOR inhibitors, and recent proposals including biologics therapy (e.g., anti-EGFR antibody), as well as ultrasound guided percutaneous microwaves [191]. A recent review associated with meta-analysis including a total of 262 patients in 40 studies discussed the role of topical applications/indication of mTOR inhibitors and evaluation of their efficacy and safety in dermatologic conditions including TSC [197]. The study identified amongst 11 dermatological conditions that over 157 of the 262 patients frequently had mostly angiofibromas linked to TSC, and that topically applied mTOR inhibitors, such as sirolimus was more effective against angiofibromas than placebo and were well tolerated [197]. Importantly, everolimus (Afinitor) has been approved by FDA for treatment of certain types of brain and kidney tumors caused by TSC [198], and there is more hope in future to target this to clearing of cutaneous tumor-like lesion and systemic disease management.

## 7. Clinical Implications, Conclusions, and Future Prospects

The identification of disease molecular basis and targets for most cutaneous malignancies are well-understood and possesses diagnostic and therapeutic indications, as it will permit the development of novel, safer, cheaper, as well as effective delivery of chemotherapeutic, biologic, and natural dietary agents targeting the dysfunction towards maintaining skin tissue homeostasis and integrity. Given that the PI3K/Akt/mTOR signaling plays a crucial role in skin cancers, targeting this pathway with therapeutic nature-derived bioactive phytochemicals, biologic molecules, and synthetic small molecules alone or in various combinations is a promising strategy to treat skin cancers. So far, several of the aforementioned synthetic molecules have been widely employed as prescription agents in clinical trials. Most of these agents have known limitations as undesired side effects, and thus require the careful selection and development of more potent, cost effective, and safer remedies [199,200,201]. Natural phytochemicals and synthetic molecules have been extensively tested for their ability to remedy PI3K/Akt/mTOR-associated cutaneous disorders [202,203]. While the global market for nutraceuticals research was estimated to reach approximately $340 billion by 2024 (Variant Market Research, Pune, India), nothing is yet suggestive of the synthetics and their analogs. Recent technological innovations in in vitro and in vivo animal disease models coupled with high throughput drug screening will speed up anticancer drug discovery and development. Some inhibitors currently available are being tested in early-stage clinical trials, and their applications in the treatment of skin related malignancies warrant further testing.

## 8. Materials and Methods

A literature search was performed in PubMed using the following MESH terms (“melanoma” OR “skin” OR “cancer small molecule”) AND (“mTOR, PI3K, Akt or mTOR, PI3K/Akt inhibitors” OR “phytochemical and skin cancer” OR “Merkel cell carcinoma and PI3K/Akt/mTOR”) AND (“skin, cutaneous, cancer” OR “Biologics for mTOR” OR “cutaneous malignancies”). We then identified 307 reviews/original articles, observational studies and guidelines and selected relevant English-language publications based on their abstracts. Publications of all studies investigating the mechanism and the use of pathway inhibitors in dermatological malignancies were included, and the most references were targeted within the last 6 years except for very rare conditions or reports.

## Figures and Tables

**Table 1:** List of reported lead synthetic compounds targeting mTOR, PI3K, and Akt signaling pathway in various skin cancers.

Lead Compound(s)	Protein Target(s)	Skin Cancer Type	Compound Structure	References
Everolimus (RAD-001)	mTOR	Melanoma, Basal Cell Carcinoma	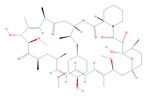	[92,93]
Erufosine	mTOR	Oral Squamous Cell Carcinoma		[94]
GDC-0084	PI3K, mTOR, Akt	cutaneous Squamous Cell Carcinoma		[95]
Isoselenocyanate-4	Akt	Melanoma	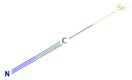	[96]
MLN0128 (Sapanisertib)	mTOR	Melanoma Merkel Cell Carcinoma	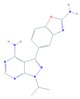	[97,98,99]
NVP-BEZ235	PI3K, Akt, mTOR	Melanoma Merkel Cell Carcinoma	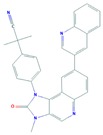	[100,101]
NC1 domain of collagen Type XIX [NC1(XIX)]	PI3K, Akt, mTOR, FAK	Melanoma		[89,90,91]
PBISe	Akt3	Invasive metastatic Melanoma	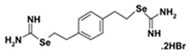	[102]
Rapamycin	PI3k, Akt, mTOR	Melanoma, Esophageal squamous cell carcinoma	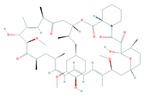	[77,78,103]
SKLB-M8	Akt, mTOR	Melanoma		[87,88]
PI-103	PI3K, mTOR	Melanoma		[104]
Perifosine	Akt	Metastatic Melanoma		[105]
Tazarotene	IGFR, PI3K, Akt, mTOR	Basal cell carcinoma		[106,107]
Temsirolimus	mTOR	Metastatic melanoma	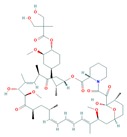	[108]
WYE-354	mTOR	Merkel cell carcinoma		[109]
VS-5584	PI3K and mTOR	Melanoma		[67,84,85,86]
Itraconazole	PI3K and mTOR	Melanoma Basal Cell Carcinoma		[110,111]
LY3023414	PI3K/mTOR	Cutaneous Basal Cell Carcinoma, cutaneous Squamous Cell Carcinoma		[112]
Ku-0063794	mTORC1 and mTORC2	BRAF-Mutant Melanoma in combination with MEK inhibitory agents Merkel cell carcinoma	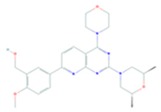	[113]

**Table 2:** List of reported lead natural dietary compounds and extracts targeting mTOR, PI3K, and Akt signaling pathway in various skin cancers.

Lead Compound (s)	Protein Targets	Skin Cancer Type	Compound Structure	References
Acacetin	PI3K, Akt, mTOR	Malignant Melanoma	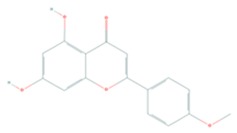	[124]
Bee Venom Melittin	PI3K, Akt, mTOR	Melanoma		[129]
Capsaicin	PI3K, Akt, Rac1	Melanoma	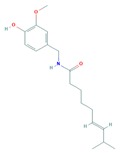	[126]
Curcumin	PI3K, Akt, mTOR	Melanoma	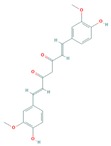	[142,143]
Epigallocatechin-3 (EGCG)	mTOR	Melanoma	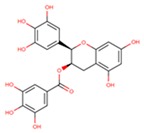	[148]
Evodiamine	PI3K, Akt	Melanoma	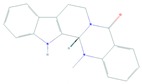	[127]
Fisetin	PI3K, Akt, mTOR	Melanoma	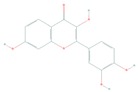	[68,139,140,141]
Isoliquiritigenin	mTORC2, Akt, GSK-3β	Melanoma cachexia	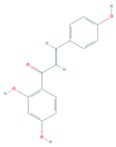	[128]
Harmine	Akt, mTOR and ERK1/2	Melanoma	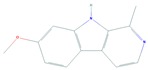	[131]
Obatoclax	Akt, mTOR	Melanoma		[133]
Panduratin A	mTOR	Melanoma	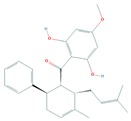	[130]
Prodigiosin	Akt, mTOR	Melanoma		[133]
Resveratrol	Akt, mTOR	Melanoma	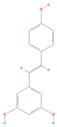	[46,144,145,146]
Sinomenine	PI3K, Akt, mTOR	Melanoma	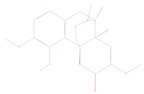	[132]
Honokiol	mTOR	Melanoma, Oral squamous cell carcinoma	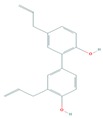	[63,147,151]
Nexrutine^R^	PI3K/Akt/mTOR	Melanoma		[149,150]

## Abbreviations

PI3K	phosphatidyl-inositiol 3-kinase
BMZ	Bbaement membrane zone
mTOR	mammalian target of rapamycin
UV	ultraviolet
EGCG	Epigallocatechin-3-gallate
Akt	protein kinase B
NMSC	non-melanoma skin cancer
SCC	squamous cell carcinoma
BCC	basal cell carcinoma
MCC	Merkel cell carcinoma
K6	keratin 6
K14	keratin 14
EMT	epithelial-mesenchymal transition
CK15	cytokeratin 15
CK5	cytokeratin
